# Factors affecting breastfeeding practices in Yunnan Province, China: data from cross-sectional national household health services surveys in 2008, 2013, and 2018

**DOI:** 10.3389/fpubh.2025.1635513

**Published:** 2025-09-18

**Authors:** Qiong Zhang, Danyang Li, Yayun Tan, Puxian Peng, Karthikesu Kartheepan, Songyuan Tang

**Affiliations:** ^1^Department of Obstetrics and Reproductive Medicine, Affiliated Hospital of Yunnan University, Kunming, Yunnan, China; ^2^Institute of Health Studies, School of Public Health, Kunming Medical University, Kunming, Yunnan, China; ^3^Institute of Health Studies, School of Medicine, Nankai University, Tianjin, China; ^4^Department of Primary Health Care, Faculty of Health-Care Sciences, Eastern University, Chenkalady, Sri Lanka; ^5^Yunnan Health and Development Research Association, Kunming, Yunnan, China

**Keywords:** breastfeeding, prevalence, factors, Yunnan, maternal health

## Abstract

**Background:**

Breastfeeding is essential for infant health and maternal well being, yet China continues to experience relatively low breastfeeding rates, with significant regional disparities. Yunnan Province, characterized by its unique demographic and geographic profile, may face even greater challenges. This study aims to investigate the prevalence and factors of breastfeeding practices in Yunnan Province using data from the 2008, 2013, and 2018 National Household Health Services Surveys (NHHSS).

**Methods:**

This cross-sectional study included 742 mother-child pairs in 2008, 909 in 2013, and 620 in 2018. We assessed key breastfeeding indicators, including overall breastfeeding rate, early initiation of breastfeeding, exclusive breastfeeding for 6 months or longer, continued breastfeeding for at least 1 year, and timely introduction of complementary foods. Multivariate logistic regression was used to analyze the associations between breastfeeding practices and maternal, child, and health service-related factors.

**Results:**

The overall breastfeeding rate in 2018 was 94.35%, with no significant difference across the three survey years (*P* > 0.05). Early initiation of breastfeeding increased from 33.83% in 2008 to 41.61% in 2018. Continued breastfeeding rose from 0.22% in 2013 to 7.10% in 2018. Urban mothers were more likely to initiate breastfeeding early but less likely to practice exclusive or continued breastfeeding (OR = 0.684, 95% CI: 0.532–0.879, *P* < 0.05). Older maternal age was associated with lower rates of continued breastfeeding (OR = 0.453, 95% CI: 0.227–0.904, *P* < 0.05). Han ethnicity, adequate antenatal care, and postpartum visits were positively associated with continued breastfeeding (*P* < 0.05). Mothers without chronic conditions were more likely to practice breastfeeding and introduce complementary foods on time.

**Conclusion:**

While the overall breastfeeding rate in Yunnan Province is relatively high, the rates of exclusive and continued breastfeeding remain low. The findings highlight the need for targeted interventions and stronger social and health system support to promote sustained breastfeeding practices. Enhanced public education and maternal support services are crucial for improving breastfeeding outcomes in the region.

## Introduction

Breastfeeding is crucial for a child's health because it gives them energy and essential nutrients for the rest of their lives, reducing their risk of death and preventing malnutrition, obesity, and hunger (1). The World Health Organization advises starting breastfeeding as soon as possible after delivery, continuing it exclusively for the first 6 months of life, and continuing it for up to 2 years or longer with appropriate and safe supplemental meals. China has been aggressively pushing the adoption of breastfeeding habits over the past few decades. The National Program of Action for Child Development in China, which runs from 2011 to 2020, has a new goal: by the time a child reaches the 6th month of life, 50% of them will have exclusively breastfed.

However, breastfeeding practices in China are generally rather inadequate, leaving much to be desired. According to data from China's Under-5 Child Nutrition and Health Surveillance System, the country's 2018 rates of exclusive breastfeeding for at least 6 months and of breastfeeding initiation within the first hour of birth are both significantly lower than the global target of a 50% exclusive breastfeeding for 6 months or more (EBF) rate at 6 months by 2025 (2). There are also substantial regional differences in China's breastfeeding rates, according to recent data. For example, “any breastfeeding” rates varied from 43.13 to 95.50% in cohort studies carried out in 17 Chinese cities (3). A 2020 study covering 26 provinces (*n* = 1,001) found that 51.6% of children were breastfed for ≥12 months. CNHS 2013 data reported an exclusive breastfeeding rate at 6 months of 20.8%, and continued breastfeeding rates of 11.5% at 1 year and 6.9% at 2 years (4).

In order to design breastfeeding promotion methods in China, it is imperative to investigate the prevalence and factors of breastfeeding, considering the nation's presently low breastfeeding rates and significant regional variances in breastfeeding rates.

There is a lack of study on breastfeeding in ethnic minority areas in the southwest of the country, with the majority of studies concentrated on economically developed coastal and Han Chinese regions. The multiethnic and less developed province of Yunnan is located in southwest China. The region's poverty, ethnic diversity, and accessibility may provide unique difficulties for maintaining breastfeeding practices. Therefore, this study aims to examine factors affecting breastfeeding practices in Yunnan by using information from the province survey carried out in Yunnan Province in 2008, 2013, and 2018, this study examined a number of factors that influence breastfeeding practices in the region, such as exclusive breastfeeding at 6 months, continuing breastfeeding at a year, prolonging breastfeeding, and introducing complementary foods on schedule.

## Methods

### Study design

This study is a repeated cross-sectional survey analyzing data from the National Health Service Survey (NHSS) conducted in Yunnan Province in 2008, 2013, and 2018.

### Data source

Based on information from the National Health Service Survey (NHSS), the National Household Health Survey (NHHS) was carried out in 2008, 2013, and 2018. The NHSS provides vital data to the government about the health of both urban and rural populations, the use of health services, and the costs and burdens associated with health care.

Analytical studies on key populations, including women and children, have been introduced since the fourth NHSS. Multistage stratified random cluster sampling was used for the NHHS. In the first stratum, systematic sampling was used to select 10 counties (districts, municipalities) from the entire province (five from each of the urban and rural areas); in the second stratum, quota (economic, geographic) simple random sampling was used to select five towns from each county (district, city); in the third stratum, quota (economic, geographic) simple random sampling was also used to select two villages (neighborhood committees) from each town; and in the fourth stratum, systematic sampling was employed to select 60 sample households from each village (neighborhood committee).

This study includes data on general basic family situations, personal situations of family members, basic situations of females aged 15–64, and basic personal characteristics of children under the age of 5 (4–6).

The survey was conducted using paper-based structured questionnaires administered face-to-face by trained interviewers, rather than self-administered questionnaires, to ensure participation from mothers with low literacy levels, particularly in ethnic minority areas of Yunnan. All interviewers were health workers or trained investigators who had received standardized training before the survey. Responses were recorded on paper questionnaires, and double data entry with cross-checking was performed for quality control.

### Participants

A total of 20,803 participants were sampled in 2008, 20,225 in 2013, and 19,490 in 2018 over the three surveys. Women who completed the basic infant profile section of the survey and had at least one live delivery within 5 years during each survey round (*n* = 4,278 in 2008, *n* = 6,777 in 2013, and *n* = 6,167 in 2018) were included in this study. In order to get data on breastfeeding practices among women of childbearing age, surveys of children under five were also conducted (*n* = 1,150 in 2008, *n* = 1,354 in 2013, and *n* = 1,735 in 2018). The surveys only inquired about the child's most recent birth within the previous 5 years.

The children's survey was administered to the mother of the child, and therefore only mother–child pairs were included. Children who were not primarily cared for by their mothers (e.g., left-behind children under the care of grandparents or other relatives) were not captured in the survey. Five mother-child pairs with repeated measures were deleted, but the most current record was kept in consideration of the likelihood of repeated measurements on the same participant in each sample. Ultimately, there were 742, 909, and 620 mother-child pairs in the surveys conducted in 2008, 2013, and 2018, respectively.

### Variables

Practiced breastfeeding, early breastfeeding initiation, exclusive breastfeeding for 6 months or more (EBF), continuous breastfeeding for a year or more (CBF), and timely introduction of supplementary foods were among the dependent variables.

The WHO recommendations (1) were followed in this study's definition of the five outcome indicators, which are as follows: any breastfeeding refers to feeding the baby with breast milk at least once after delivery; early initiation of breastfeeding refers to feeding the baby with breast milk within the first hour of life; and exclusive breastfeeding (EBF) refers to feeding the baby only breast milk for at least 6 months after birth, without the addition of any solid foods or liquids, including water, vitamins, or minerals. When a child receives enough supplemental feedings in addition to breast milk for a year or more, it is referred to as continuous breastfeeding (CBF). When complementary meals are introduced on schedule, the child is weaned entirely from breast milk for 6 months, at which point supplemental foods are introduced.

The indicators of the dependent variable surveyed each year are shown in Table 1.

**Table 1:** Dependent variables indicators for each survey.

**Breastfeeding practices**	**2008**	**2013**	**2018**
Practiced any breastfeeding	√	√	√
Early initiation of breastfeeding (EIBF)	√		√
Exclusive breastfeeding for 6 months or more (EBF)		√	√
Continued breastfeeding for 1 year or more (CBF)		√	√
Timely introduction of complementary food		√	√

The characteristics of the mother, the child, and the health services for children 0–5 years old are independent variables in this study. The number of visits within 42 days after delivery (0, 1, and ≥2), mode of delivery (vaginal delivery, cesarean section), place of delivery (county and above hospitals, maternal and child health care facilities, others township health centers, health service centers, home and clinics, or other), and frequency of prenatal checkups (< 5, 5–7, and ≥8) were among the variables based on health service characteristics. Mother-based factors included parity (1, ≥2), household wealth (poorest, middle, and richest), age at delivery (< 25, 25–34, and ≥35), ethnicity (other, Han), region (rural, urban), educational level (illiterate or primary school, secondary or high school, bachelor's degree or above), occupation (employed, unemployed, or underemployed), and whether they had a chronic illness and whether they felt depressed or anxious. Age (in months), gender, and birth weight (low birth weight: < 2,500 g, normal weight: 2,500–4,000 g, and macrosomia: >4,000 g) were the variables based on children aged 0–5 years.

### Data analysis

For descriptive analyses, participant characteristics across the three survey years—for mothers, children aged 0–5 years, and health services—were summarized as follows: categorical variables by frequencies and percentages, and continuous variables by means and standard deviations. Descriptive statistics were used in this study to describe the baseline characteristics of the participants and breastfeeding practices in 2008, 2013, and 2018.

For categorical data, chi-square tests were used, while *U*-tests were used for continuous variables. By dividing the data from the three surveys (2008, 2013, and 2018) by characteristics related to mothers, children, and health services, as well as the survey year, chi-square tests were utilized to evaluate differences between baseline characteristics and investigate rates and variations in various breastfeeding practices. Explanatory variables such as survey year, mother-based, child-based, and healthcare-based characteristics were analyzed using bivariate and multivariate logistic regressions to investigate the association between breastfeeding practices and these factors.

After adjusting for potential confounders, multivariate logistic regressions were performed to investigate associations with early initiators of breastfeeding (EIBF) outcomes. The models controlled for maternal characteristics (age at delivery, ethnicity, region of residence, educational level, parity, household wealth, occupation, chronic disease, anxiety or depression), health service characteristics (number of antenatal visits, mode of delivery, place of delivery, and postpartum visits), and child characteristics (age, gender, and birth weight). For the 2008 and 2018 data, univariate analyses were performed to assess differences in EIBF across these maternal, health service, and child characteristics, estimating crude odds ratios (ORs) and 95% confidence intervals (CIs).

After combining the databases from 2013 and 2018, confounders were controlled for, and multivariate logistic regression was used to investigate the factors that influence each of the five breastfeeding practices. Each logistic regression model reported adjusted ORs and 95% CIs, and a *P* < 0.05 was considered statistically significant. The data analysis was done using SPSS 26.0.

## Results

### Socio-demographic characteristics

This study included 2,271 mother-child pairs in total (*n* = 742 in 2008, 909 in 2013, and 620 in 2018). Table 2 displays the participant characteristics according to children, mothers, and health services. The age at which women gave birth increased from 26.04 ± 4.537 years in the 2008 survey to 27.78 ± 6.025 years in the 2018 survey (*P* < 0.001), with mothers in the 25–34 year age group making up the largest percentage of mothers in all three surveys.

**Table 2:** Characteristics of surveyed mother-infant pairs.

**Characteristics**	**2018 N (%)**	**2013 N (%)**	**2008 N (%)**	**χ2**	***P*-values**
**Health services' characteristics**					
**Antenatal visits**					
0–4	90 (14.50)	361 (39.70)	377 (50.80)	464.527	**< 0.001**
5–7	149 (24.00)	401 (44.10)	214 (28.80)		
≥8	381 (61.50)	147 (16.20)	151 (20.40)		
**Place of delivery**					
County- or higher-level hospital	359 (57.90)	386 (42.50)	436 (58.80)	115.391	**< 0.001**
Maternal and Child Health Hospital	188 (30.30)	239 (26.30)	122 (16.40)		
Primary hospital/private hospital/home, clinic or other	73 (11.80)	284 (31.20)	184 (24.80)		
**Mode of delivery**					
Vaginal	455 (73.40)	709 (78.00)	571 (77.00)	4.535	0.104
Cesarean section	165 (26.60)	200 (22.00)	171 (23.00)		
**Postpartum visits**					
0	220 (35.50)	357 (39.30)	326 (43.90)	94.048	**< 0.001**
1	224 (36.10)	175 (19.30)	118 (15.90)		
≥2	176 (28.40)	377 (41.50)	298 (40.20)		
**Mothers' characteristics**					
Age, years ($\bar{x}\pm S$)	27.78 ± 6.025	25.45 ± 4.841	26.04 ± 4.537	86.699	**< 0.001**
≤ 25	194 (31.30)	423 (46.50)	302 (40.70)		
25 to < 35	334 (53.90)	438 (48.20)	409 (55.10)		
≥35	92 (14.80)	48 (5.30)	31 (4.20)		
**Ethnicity**					
Minority	262 (42.30)	299 (32.90)	210 (28.30)	30.093	**< 0.001**
Han Chinese	358 (57.70)	610 (67.10)	532 (71.70)		
**Region**					
Urban	301 (48.50)	386 (42.50)	256 (34.50)	28.000	**< 0.001**
Rural	319 (51.50)	523 (57.50)	486 (65.50)		
**Maternal education**					
Junior school or below	219 (35.30)	404 (44.40)	302 (40.70)	54.252	**< 0.001**
Senior high school	293 (47.30)	444 (48.80)	379 (51.10)		
Bachelor degree or above	108 (17.40)	61 (6.70)	61 (6.70)		
**Occupation**					
Employed	491 (79.20)	852 (93.70)	643 (86.70)	71.595	**< 0.001**
Unemployed	129 (20.80)	57 (6.30)	99 (13.30)		
**Depression or Anxiety**					
Yes	39 (6.30)	27 (3.00)	26 (3.50)	11.301	**0.004**
No	581 (93.70)	882 (97.00)	716 (96.50)		
**Chronic disease**					
Yes	53 (8.50)	28 (3.10)	25 (3.40)	28.942	**< 0.001**
No	567 (91.50)	881 (96.90)	717 (96.60)		
**Household wealth**					
Poorest	97 (15.60)	148 (16.30)	334 (45.00)	298.132	**< 0.001**
Middle	137 (22.10)	248 (27.30)	225 (30.30)		
Richest	386 (62.30)	513 (56.40)	183 (24.70)		
**Parity**					
Primiparous	199 (32.10)	446 (49.10)	438 (59.00)	99.365	**< 0.001**
Multiparous	421 (67.90)	463 (50.90)	304 (41.00)		
**Children's characteristics**					
**Age, months**					
0 to < 6	66 (10.60)	30 (3.30)	20 (2.70)	**55.116**	**< 0.001**
6 to < 12	166 (26.80)	281 (30.90)	233 (31.40)		
≥12	388 (62.60)	598 (65.80)	489 (65.90)		
**Gender**					
Male	320 (51.60)	476 (52.40)	378 (50.90)	0.333	0.847
Female	300 (48.40)	433 (47.60)	364 (49.10)		
**Birth weight, %**					
Low birth weight (< 2,500 g)	28 (4.50)	28 (3.10)	19 (2.60)	14.443	**0.006**
Normal birth weight (2,500–3,999 g)	545 (87.90)	818 (90.00)	694 (93.50)		
Macrosomia (≥4,000 g)	47 (7.60)	63 (6.90)	29 (3.90)		

Bold values represent statistically significant differences (*P* < 0.05).

Han Chinese mothers made up 57.70% of all mothers in 2018, a decrease from 71.70% in 2008. The percentage of the population that lives in urban went from 34.50 to 48.50% (*P* < 0.001). About 50% of the mothers who participated in the study had completed high school. The proportion of mothers with anxiety or depression increased (*P* < 0.05) and the percentage of mothers with chronic disorders increased from 3.4 to 8.5% in 2018 compared to mothers in 2008. The mean yearly household income increased to 49,444 yuan (*P* < 0.001) from 17,018 yuan. In 2008, 41.0% of mothers had more than two children; by 2018, that number had risen to 67.90%.

When comparing 2018–2008 and 2013, the percentage of prenatal visits with a frequency of ≥8 visits increased significantly (*P* < 0.001). Between 2008 and 2018 (*P* < 0.001), the percentage of 42-day postpartum visits with zero frequency decreased. The majority of the children had normal birth weights and were older than 12 months.

### Breastfeeding practices

Breastfeeding practices are illustrated in Table 3. There was no difference in the overall breastfeeding rate in Yunnan Province among the three surveys (*P* > 0.05), and the rate of breastfeeding in 2018 was 94.35%.

**Table 3:** Breastfeeding practices in 2008, 2013 and 2018 in Yunnan Province, China.

**Breastfeeding practices**	**2018, %b**	**2013, %b**	**2008, %b**	**χ2**	***P-*values**
**Timing of breastfeeding initiation**					
Within 30 min after birth	177 (28.55)	–	155 (20.89)	18.675	**< 0.001**
Above 30 min to 1 h after birth	81 (13.06)	–	96 (12.94)		
Above 1 to 24 h after birth	138 (22.26)	–	233 (31.40)		
The second day after birth and later	189 (30.48)	–	217 (29.25)		
Never	35 (5.65)	47 (5.17)	41 (5.53)		
Early initiation of breastfeeding	258 (41.61)	–	251 (33.83)	9.179	**0.002**
Exclusive breastfeeding for 6 months or more	262 (42.26)	397 (43.67)	–	0.226	0.634
Continued breastfeeding for 1 year or more	44 (7.10)	2 (0.22)	155 (20.89)	60.161	**< 0.001**

Bold values represent statistically significant differences (*P* < 0.05).

To clarify timing indicators: we report two early-initiation measures—initiation within 30 min after birth (a measure of very-early initiation reflecting immediate delivery-room practices) and initiation within 1 h after birth (the WHO-standard “early initiation” indicator). In 2018, the proportion of infants who initiated breastfeeding within 30 min was 28.55%, compared with 20.89% in 2008. The proportion of infants who initiated breastfeeding within 1 h increased from 33.83% in 2008 to 41.61% in 2018.

There was no significant difference in the proportion of EBF between 2013 and 2018 (*P* > 0.05). The number of people with CBF was 7.10% in 2018 and only 0.22% with CBF in 2013. The percentage of CBF in 2018 was significantly higher than that in 2013 (*P* < 0.001).

### Factors of breastfeeding practices

The factors that influence EIBF in 2008 and 2018 are shown in Table 4. Mothers who had cesarean sections (*P* < 0.001), resided in urban areas (*P* < 0.05), were nervous or depressed (*P* < 0.05), gave birth in a primary hospital, private hospital, home, clinic, or another location (*P* < 0.05) were more likely to start breastfeeding early (Table 3). Table 3 shows a negative correlation between the number of postpartum visits (*P* < 0.05) and the birth weight of a baby, which can be either normal or overweight, and the early commencement of breastfeeding.

**Table 4:** Factors of early initiation of breastfeeding in 2008 and 2018 in Yunnan Province, China.

**Characteristics**	**2008**			**2018**		
	**Crude OR (95% CI)**	**Adjusted OR (95% CI)**	***P-*** **value**	**Crude OR (95% CI)**	**Adjusted OR (95% CI)**	***P*** **-value**
**Mothers' characteristics**						
**Age at delivery (year)**						
< 25 (Ref.)	1.000	1.000		1.000	1.000	
25–34	1.085 (0.717, 1.641)	1.155 (0.451, 2.955)	0.764	1.426 (0.846, 2.402)	1.557 (0.817, 2.971)	0.179
≥35	1.406 (0.895, 2.207)	1.088 (0.446, 2.653)	0.852	1.259 (0.773, 2.052)	1.362 (0.781, 2.374)	0.276
**Ethnicity**						
Minority (Ref.)	1.000	1.000		1.000	1.000	
Han	1.168 (0.831, 1.641)	1.183 (0.800, 1.751)	0.399	1.461 (1.050, 2.033)	1.062 (0.713, 1.584)	0.766
**Region**						
Rural (Ref.)	1.000	1.000		1.000	1.000	
Urban	1.699 (1.212, 2.383)	1.555 (0.971, 2.489)	0.066	1.951 (1.400, 2.719)	**2.163 (1.401, 3.339)**	**0.001**
**Educational level**						
Illiterate or primary school(Ref.)	1.000	1.000		1.000	1.000	
Secondary or high school	2.098 (1.034, 4.258)	1.193 (0.475, 2.997)	0.707	1.487 (0.920, 2.405)	0.630 (0.332, 1.194)	0.157
Bachelor's degree or above	2.259 (1.124, 4.543)	1.395 (0.601, 3.240)	0.438	1.260 (0.796, 1.996)	0.828 (0.485, 1.413)	0.49
**Parity**						
1 (Ref.)	1.000	1.000		1.000	1.000	
≥2	0.841 (0.615, 1.149)	0.963 (0.636, 1.458)	0.858	0.638 (0.447, 0.911)	**0.589 (0.374, 0.929)**	**0.023**
**Household wealth**						
Poorest (Ref.)	1.000	1.000		1.000	1.000	
Middle	1.219(0.821, 1. 808)	0.900 (0.550, 1.473)	0.675	0.919 (0.577, 1.463)	0.705 (0.405, 1.226)	0.216
Richest	1.230 (0.805, 1.879)	0.977 (0.601, 1.590)	0.927	1.596 (1.068, 2.386)	1.133 (0.710, 1.807)	0.601
**Occupation**						
Employed (Ref.)	1.000	1.000		1.000	1.000	
Unemployed	1.853 (1.115, 3.079)	1.363 (0.747, 2.486)	0.313	1.622 (1.061, 2.480)	1.565 (0.956, 2.561)	0.075
**Chronic disease**						
Yes (Ref.)	1.000	1.000		1.000	1.000	
No	0.488 (0.179, 1.330)	0.473 (0.164, 1.364)	0.166	0.569 (0.301, 1.075)	0.605 (0.299, 1.227)	0.164
**Anxiety or Depression**						
No (Ref.)	1.000	1.000		1.000	1.000	
Yes	2.872 (0.971, 8.499)	**3.388 (1.087, 10.555)**	**0.035**	0.731 (0.369, 1.449)	0.605 (0.277, 1.318)	0.206
**Health services characteristics**						
**Antenatal visits**						
< 5 (Ref.)	1.000	1.000		1.000	1.000	
5–7	1.085 (0.717, 1.641)	0.847 (0.484, 1.482)	0.561	2.033 (1.261, 3.279)	1.978 (1.121, 3.489)	0.019
≥8	1.406 (0.895, 2.207)	1.048 (0.619, 1.774)	0.862	1.567 (1.056, 2.326)	1.341 (0.845, 2.128)	0.213
**Mode of delivery**						
Vaginal delivery (Ref.)	1.000	1.000		1.000	1.000	
Cesarean section	**2.821 (1.839, 4.329)**	**3.455 (2.132, 5.596)**	**< 0.001**	2.683 (1.789, 4.023)	**2.416 (1.543, 3.785)**	**< 0.001**
**Place of delivery**						
County or higher-level hospital (Ref.)	1.000	1.000		1.000	1.000	
Maternal and Child Health hospital	1.257 (0.858, 1.840)	1.592 (0.972, 2.608)	0.065	1.034 (0.605, 1.767)	1.383 (0.746, 2.564)	0.304
Primary hospital/private hospital/home, clinic, or other	1.757 (1.081, 2.855)	1.519 (0.885, 2.609)	0.129	1.429 (0.807, 2.528)	**2.585 (1.337, 5.000)**	**0.005**
**Postpartum visits**						
0 (Ref.)	1.000	1.000		1.000	1.000	
1	**0.442 (0.314, 0.622)**	**0.468 (0.317, 0.692)**	**0.013**	1.077 (0.709, 1.634)	1.011 (0.635, 1.610)	0.962
≥2	**0.528 (0.332, 0.841)**	**0.524 (0.315, 0.872)**	**< 0.001**	1.312 (0.869, 1.981)	1.277 (0.812, 2.008)	0.289
**Children's characteristics**						
**Age, months**						
≤ 6 (Ref.)	1.000	1.000		1.000	1.000	
6–12	1.334 (0.526, 3.383)	1.886 (0.670, 5.307)	0.230	0.676 (0.389, 1.175)	0.856 (0.463, 1.579)	0.618
>12	1.048 (0.750, 1.465)	1.069 (0.738, 1.550)	0.723	0.879 (0.601, 1.286)	1.092 (0.710, 1.678)	0.689
**Gender**						
Male (Ref.)	1.000	1.000		1.000	1.000	
Female	0.765 (0.562, 1.043)	0.773 (0.555, 1.078)	0.129	0.938 (0.677, 1.300)	0.969 (0.679, 1.382)	0.862
**Birth weight**						
< 2,500 (Ref.)	1.000	1.000		1.000	1.000	
2,500–4,000	0.862 (0.256, 2.894)	0.468 (0.120, 1.827)	0.275	0.688 (0.254, 1.864)	**0.322 (0.107, 0.972)**	**0.044**
>4,000	0.671 (0.317, 1.418)	**0.398 (0.170, 0.933)**	**0.034**	1.048 (0.570, 1.926)	0.657 (0.332, 1.299)	0.227

The models were adjusted for maternal characteristics (age at delivery, ethnicity, region, educational level, parity, household wealth, occupation, chronic disease, anxiety or depression), health service characteristics (antenatal visits, mode of delivery, place of delivery, postpartum visits), and child characteristics (age in months, gender, birth weight). Bold values represent statistically significant differences (*P* < 0.05).

Table 5 displays the correlation between breastfeeding practices between 2013 and 2018 and motherhood, health services, and child characteristics. Breastfeeding rates were found to be lower for mothers who were unemployed or unemployable (OR = 0.293, 95% CI :0.169, 0.508, *P* < 0.001), lower for women who had a cesarean section (OR = 0.503, 95% CI: 0.0.299, 0.844, *P* < 0.05), and higher for mothers without a chronic illness (OR = 3.304, 95% CI: 1.524, 7.161, *P* < 0.05).

**Table 5:** Logistic regressions of breastfeeding measures and demographic and socioeconomic characteristics. (Data from 2008 to 2018).

**Characteristics**	**Practiced any breastfeeding**				**Exclusive breastfeeding for 6 months or more (EBF)**				**Continued breastfeeding for 1 years or more (CBF)**				**Timely introduction of complementary food**			
	**OR**	**95%CI**		***P-*** **value**	**OR**	**95%CI**		***P-*** **value**	**OR**	**95%CI**		***P-*** **value**	**OR**	**95%CI**		***P-*** **value**
**Mothers' characteristics**																
**Age at delivery (year)**																
< 25 (Ref.)	1.000				1.000				1.000				1.000			
25–34	0.963	0.387	2.395	0.935	0.880	0.571	1.356	0.562	**0.312**	**0.143**	**0.682**	**0.004**	0.77	0.371	1.6	0.484
≥35	0.932	0.412	2.108	0.865	0.825	0.557	1.222	0.336	**0.453**	**0.227**	**0.904**	**0.025**	1.07	0.56	2.048	0.837
**Ethnicity**																
Minority (Ref.)	1.000				1.000				1.000				1.000			
Han	0.59	0.336	1.036	0.066	0.962	0.759	1.221	0.751	**1.822**	**1.117**	**2.973**	**0.016**	0.958	0.619	1.483	0.848
**Region**																
Rural (Ref.)	1.000				1.000				1.000				1.000			
Urban	0.727	0.426	1.241	0.243	**0.684**	**0.532**	**0.879**	**0.003**	**0.236**	**0.137**	**0.405**	**< 0.001**	1.404	0.896	2.200	0.139
**Educational level**																
Illiterate or primary school (Ref.)	1.000				1.000				1.000				1.000			
Secondary or high school	1.452	0.583	3.616	0.423	0.914	0.596	1.402	0.680	1.827	0.666	5.01	0.242	1.034	0.512	2.091	0.925
Bachelor degree or above	1.105	0.486	2.514	0.812	0.932	0.636	1.365	0.717	2.236	0.87	5.748	0.095	1.495	0.803	2.781	0.205
**Parity**																
1 (Ref.)	1.000				1.000				1.000				1.000			
≥2	1.384	0.806	2.377	0.238	1.094	0.852	1.405	0.482	1.102	0.652	1.864	0.716	0.982	0.625	1.544	0.938
**Household wealth**																
Poorest (Ref.)	1.000				1.000				1.000				1.000			
Middle	1.118	0.568	2.200	0.747	1.236	0.898	1.700	0.193	1.268	0.654	2.457	0.482	0.430	0.799	0.458	1.395
Richest	0.766	0.408	1.437	0.406	0.907	0.692	1.188	0.476	1.021	0.595	1.753	0.940	0.841	1.053	0.635	1.747
**Occupation**																
Employed (Ref.)	1.000				1.000				1.000				1.000			
Unemployed	**0.293**	**0.169**	**0.508**	**< 0.001**	0.736	0.521	1.040	0.083	1.061	0.509	2.210	0.875	**2.512**	**1.562**	**4.04**	**< 0.001**
**Chronic disease**																
Yes (Ref.)	1.000				1.000				1.000				1.000			
No	**3.304**	**1.524**	**7.161**	**0.002**	0.814	0.487	1.359	0.431	0.536	0.125	2.296	0.401	**0.440**	**0.216**	**0.894**	**0.023**
**Anxiety or Depression**																
No (Ref.)	1.000				1.000				1.000				1.000			
Yes	0.919	0.321	2.633	0.875	0.899	0.523	1.546	0.701	1.737	0.484	6.227	0.397	1.101	0.442	2.739	0.836
**Health services' characteristics**																
**Antenatal visits**																
< 5 (Ref.)	1.000				1.000				1.000				1.000			
5–7	1.409	0.703	2.824	0.334	**1.703**	**1.255**	**2.312**	**0.001**	**3.006**	**1.598**	**5.654**	**0.001**	0.975	0.553	1.719	0.929
≥8	1.738	0.952	3.171	0.072	**1.329**	**1.008**	**1.752**	**0.044**	1.828	0.966	3.461	0.064	0.944	0.578	1.542	0.818
**Mode of delivery**																
Vaginal delivery (Ref.)	1.000				1.000				1.000				1.000			
Cesarean section	**0.503**	**0.299**	**0.844**	**0.009**	1.011	0.776	1.318	0.935	0.877	0.502	1.530	0.643	**1.767**	**1.141**	**2.735**	**0.011**
**Place of delivery**																
County-or higher-level hospital (Ref.)	1.000				1.000				1.000				1.000			
Maternal and Child Health hospital	0.811	0.431	1.524	0.514	0.768	0.569	1.036	0.084	0.714	0.383	1.330	0.289	1.087	0.625	1.889	0.768
Primary hospital/private hospital/home, clinic or other	0.485	0.23	1.021	0.057	0.989	0.714	1.370	0.948	0.888	0.475	1.663	0.712	1.442	0.776	2.677	0.247
**Postpartum visits**																
0 (Ref.)	1.000				1.000				1.000				1.000			
1	1.114	0.646	1.924	0.697	1.082	0.837	1.398	0.547	**1.978**	**1.153**	**3.393**	**0.013**	1.152	0.720	1.843	0.555
≥2	0.792	0.422	1.484	0.466	0.903	0.685	1.192	0.472	0.831	0.416	1.660	0.600	1.095	0.676	1.773	0.713
**Children's characteristics**																
**Age, months**																
≤ 6 (Ref.)	1.000				1.000				1.000				1.000			
6–12	0.702	0.232	2.125	0.531	1.223	0.780	1.916	0.380	1.076	0.433	2.674	0.874	**0.075**	**0.043**	**0.130**	**< 0.001**
>12	1.497	0.908	2.467	0.113	0.957	0.750	1.222	0.725	**0.439**	**0.242**	**0.797**	**0.007**	**0.413**	**0.267**	**0.639**	**< 0.001**
**Gender**																
Male (Ref.)	1.000				1.000				1.000				1.000			
Female	1.239	0.777	1.977	0.367	0.897	0.725	1.109	0.316	1.001	0.645	1.552	0.998	0.985	0.671	1.448	0.940
**Birth Weight**																
< 2,500 (Ref.)	1.000				1.000				1.000				1.000			
2,500–4,000	**4.353**	**1.077**	**17.59**	**0.039**	0.678	0.336	1.368	0.278	2.347	0.710	7.758	0.162	**0.19**	**0.050**	**0.724**	**0.015**
>4,000	1.944	0.654	5.777	0.232	0.684	0.453	1.034	0.072	1.244	0.528	2.928	0.617	**0.31**	**0.107**	**0.898**	**0.031**

The models were adjusted for maternal characteristics (age at delivery, ethnicity, region, educational level, parity, household wealth, occupation, chronic disease, anxiety or depression), health service characteristics (antenatal visits, mode of delivery, place of delivery, postpartum visits), and child characteristics (age in months, gender, birth weight). Bold values represent statistically significant differences (*P* < 0.05).

Mothers living in urban areas were less likely to breastfeed for 6 months and beyond than women living in rural areas (OR = 0.684, 95% CI: 0.532, 0.879, *P* < 0.05); the number of antenatal visits was positively associated with exclusive breastfeeding for 6 months and beyond, with more antenatal visits being associated with a greater likelihood that a mother would EBF. Mothers who were older than 35 years (OR = 0.453, 95% CI: 0.227, 0.904, *P* < 0.05) and lived in urban areas (OR = 0.236, 95% CI: 0.137, 0.405, *P* < 0.001) were less likely to engage in exclusive breastfeeding for more than 1 year continuously, and women who were Han Chinese (*P* = 0.016), had several antenatal visits of 5–8 times (*P* < 0.001), and had a postpartum visit (*P* = 0.013) were more likely to breastfeed for more than 1 year.

Women without chronic diseases were less likely to add complementary foods during breastfeeding (OR = 0.440, 95% CI: 0.216, 0.894, *P* < 0.05); infants with normal birth weight (OR = 0.190, 95% CI: 0.050, 0.724, *P* < 0.05) and being overweight (OR = 0.310, 95% CI: 0.107, 0.898, *P* < 0.05) were negatively associated with the timely introduction of complementary foods during breastfeeding (Table 5). Women who were unemployed or unemployable were also more likely than employed women to add complementary foods during breastfeeding (OR = 2.512, 95% CI: 1.562, 4.040, *P* < 0.001).

## Discussion

This study, based on three provincially representative surveys in Yunnan Province, aimed to evaluate the temporal trends in breastfeeding over the past decade and their influencing factors, highlighting the significant impact of ethnicity, geographic location, and access to healthcare services on breastfeeding practices.

According to the survey, Yunnan province's exclusive breastfeeding rates in 2018 were marginally higher than the world average of 42.4% (7). The EBF rate in Yunnan Province was higher in 2008 and 2018 compared to the 27.6% rate in the fourth national health service survey conducted in 2008 (7) and the 34.9% rate in Chinese national statistics from the U5CNHSS (6). The study also revealed an increasing tendency in Yunnan province toward EIBF and CBF.

Numerous elements, including as social networks, the environment, and individual variables, have an impact on breastfeeding practices (2, 7, 8). This study found a negative correlation between the mother's urban residency and the three forms of breastfeeding that the WHO recommends. This correlation may be due to a number of reasons, including the mother's social expectations and self-efficacy. A mother's confidence in her ability to breastfeed her child is known as breastfeeding self-efficacy (5, 9).

In urban areas, the percentage of mothers with employment would likely be greater. Returning to work was a period of transition during which a number of difficulties surfaced and adversely affected the self-efficacy of breastfeeding. Several studies reported that the primary causes of formula supplementation or cessation of breastfeeding were exhaustion resulting from balancing breastfeeding and employment, as well as lack of sleep (10–12). The study found that factors influencing the decision to start breastfeeding early changed between 2008 and 2018. The primary factors in 2008 were the mother's depression and anxiety as well as the postpartum visits. It should be mentioned that in 2018, breastfeeding behaviors were shown to be influenced by parity.

Families in China were only allowed to have one child starting in 1980. In a one-child family structure, family members' high degree of breastfeeding participation may be unique to Chinese culture and society (5). Research has indicated that these attributes might potentially serve as an obstacle to breastfeeding (13, 14). China's “Comprehensive Two-Child” policy, which was put into effect in 2016, significantly altered the makeup of Chinese families. In this 2018 study, the early onset of breastfeeding was favorably correlated with parity.

The study also indicated the important role healthcare support has played in breastfeeding practices. Antenatal visits were positively correlated with both EBF and CBF rates. Meanwhile, postpartum visits were found to be positively correlated with EIBF and CBF practices. Yunnan, one of the most underdeveloped provinces in China, owned the most impoverished counties in 2014 (5, 15).

The local economy has been expanding quickly because to the National Poverty Alleviation and Development Program and the outstanding work of the government (16). As a major factor in the promotion of breastfeeding practices, the proportions of facility births, cesarean sections, antenatal visits, and postnatal visits have all reached the national norm (17).

Furthermore, the Han ethnic group and continuous breastfeeding (CBF) were shown to be positively correlated in this study. Han inhabitants as well as ethnic minorities live side by side in Yunnan, a multiethnic community (18). In the 2018 survey, ethnic minorities made up 42.3% of the sample, whilst Han mothers made up 57.7%. Ethnic minority groups in China, like those in other nations, are frequently confronted with adverse socioeconomic conditions, such as residing in rural regions distant from urban areas, experiencing poverty, and receiving less medical treatment (19–21).

Lower rates of breastfeeding failure in ethnic minority groups might be explained by a lack of information about breastfeeding practices. On the other hand, distinctive traits including history, culture, and habits of ethnic groups may also contribute to low CBF rates. For instance, the study found that the Pumi People, an ethnic community, usually stopped breastfeeding their infants at the average age of one, in accordance with custom (22, 23).

There are several strengths in this study. Initially, a systematic sampling technique was used to extract the data from three survey datasets that were provincially representative. Secondly, the variations in breastfeeding practices over the last 10 years may be seen by comparing certain results from two surveys. Third, the results of the 2018 survey provide the most recent data on breastfeeding behaviors in Yunnan Province, China, as well as proof of the significance of early breastfeeding beginnings for reaching ideal breastfeeding practices.

This study does have some limitations, though. Since Yunnan is a region with a high proportion of ethnic minorities, some mothers may be illiterate, and there may be left-behind children, which could introduce selection bias and potentially affect the study results. Some breastfeeding indicators were not comparable between the 2008 and 2018 surveys due to variations in the questions, and no comparative analysis was done. Since there were insufficient numbers of individuals who maintained breastfeeding practices for 2 years or longer in the 2013 and 2018 databases to support statistical analysis, the indicator of continued breastfeeding was not computed in accordance with the WHO criteria. In addition, the survey's questions about breastfeeding survey criteria are not all-inclusive, and they lack specificity when it comes to exclusive breastfeeding (EBF) and continued breastfeeding (CBF). This makes it more difficult to investigate the research factors for EBF and CBF further.

In summary, Yunnan breastfeeding practices may be partially explained by breastfeeding-related variables like as policy, mothers' self-efficacy, healthcare resources, and the distinctive cultures of ethnic groups. Given Yunnan's distinctive location and culture, it is advised to demand further measurements and collections in order to investigate the elements that contribute significantly to the promotion of breastfeeding practices. Furthermore, this research suggests that it would be essential for society and the government to give social assistance, improved healthcare, and appropriate education for mother's greater consideration.

## Conclusions

The current status of breastfeeding practices in Yunnan Province is relatively optimistic. However, rates of exclusive and continued breastfeeding remain suboptimal. Breastfeeding practices in Yunnan Province are influenced by factors such as policy, mothers' self-efficacy, healthcare services, and ethnic culture, and more targeted measures are needed to promote breastfeeding in the future. It is recommended to enhance breastfeeding health education, safeguard the occupational rights of female workers, and implement region-specific, targeted interventions based on local breastfeeding practices.

## Data Availability

The raw data supporting the conclusions of this article will be made available by the authors, without undue reservation.
